# Mortality Predictors in Patients with Severe Dengue in the State of Amazonas, Brazil

**DOI:** 10.1371/journal.pone.0161884

**Published:** 2016-08-26

**Authors:** Rosemary Costa Pinto, Daniel Barros de Castro, Bernardino Cláudio de Albuquerque, Vanderson de Souza Sampaio, Ricardo Augusto dos Passos, Cristiano Fernandes da Costa, Megumi Sadahiro, José Ueleres Braga

**Affiliations:** 1 Health Surveillance Foundation of Amazonas State (Fundação de Vigilância em Saúde do Amazonas, FVS), Manaus, Brazil; 2 Sérgio Arouca National School of Public Health (Escola Nacional de Saúde Pública Sérgio Arouca), FIOCRUZ, Rio de Janeiro, Brazil; 3 Laboratory of Physiology and Control of Arthropod Vectors (Laboratório de Fisiologia e Controle de Artrópodes Vetores), Instituto Oswaldo Cruz, FIOCRUZ, Rio de Janeiro, Brazil; 4 Institute of Social Medicine (Instituto de Medicina Social), Rio de Janeiro State University (Universidade do Estado do Rio de Janeiro, UERJ), Rio de Janeiro, Brazil; 5 PECTI-SAÚDE/Research Foundation of the State of Amazonas (Fundação de Amparo à Pesquisa do Estado do Amazonas, FAPEAM), Manaus, Brazil; Royal College of Surgeons in Ireland, IRELAND

## Abstract

Dengue is a major public health problem in tropical and subtropical areas worldwide. There is a lack of information on the risk factors for death due to severe dengue fever in developing countries, including Brazil where the state of Amazonas is located. This knowledge is important for decision making and the implementation of effective measures for patient care. This study aimed to identify factors associated with death among patients with severe dengue, in Amazonas from 2001 to 2013. We conducted a retrospective cohort study based on secondary data from the epidemiological surveillance of dengue provided by the Fundação de Vigilância em Saúde do Amazonas, FVS (Health Surveillance Foundation) of the Secretaria de Saúde do Amazonas, SUSAM (Health Secretariat of the State of Amazonas). Data on dengue cases were obtained from the SINAN (Notifiable Diseases Information System) and SIM (Mortality Information System) databases. We selected cases of severe dengue with laboratory confirmation, including dengue-related deaths of residents in the state of Amazonas from January 1, 2001, to December 31, 2013. The explanatory variables analyzed were sex, age, level of education, spontaneous hemorrhagic manifestations, plasma extravasation and platelet count. Patients who died due to severe dengue had more hematuria, gastrointestinal bleeding, and thrombocytopenia than the survivors. Considering the simultaneous effects of demographic and clinical characteristics with a multiple logistic regression model, it was observed that the factors associated with death were age >55 years (odds ratio [OR] 4.98), gastrointestinal bleeding (OR 10.26), hematuria (OR 5.07), and thrombocytopenia (OR 2.55). Gastrointestinal bleeding was the clinical sign most strongly associated with death, followed by hematuria and age >55 years. The study results showed that the best predictor of death from severe dengue is based on the characteristic of age >55 years, together with the clinical signs of gastrointestinal bleeding, hematuria, and low platelet count.

## Introduction

Dengue is an arbovirus recognized as a growing public health problem owing to its wide geographic dispersion and high incidence in various countries [1]. It is estimated that 390 million cases occur annually, of which 96 million display signs of severity [2], causing nearly 20,000 deaths in developing countries [3].

In Brazil, in 2014, there were 591,000 probable cases of dengue and 410 deaths from the disease [4]. In that year, 3,803 cases were confirmed and 9 deaths were reported to be caused by dengue in the state of Amazonas, where 1,658 cases were confirmed and 6 deaths occurred in its capital, Manaus [5]. In the last decade, the mortality and hospitalization rates due to dengue increased in several regions of the country [6,7].

It is noteworthy to highlight that the first dengue epidemic in the state of Amazonas occurred between the years 1998 and 1999 [8]. Since the introduction of the serotype DEN-2 in 2001, there has been an increase in the recording of severe cases and deaths from the disease [9,10]. In 2011, when the worst epidemic in the state occurred, the circulation of four serotypes was observed, with 57,805 confirmed cases and 23 deaths [11].

To reduce the mortality rate of dengue, the World Health Organization and the Ministry of Health proposed a classification of dengue cases based on a set of indicative clinical and laboratory characteristics of severity, thus allowing the recommendation of the best treatment for each situation [12,13]. While this classification might be useful for the clinical management of patients, it is known that such predictors of severity may vary according to different epidemiological settings [14,15]. In addition, the evolution to death of severe cases may be related to the presence of clinical and laboratory characteristics that are still not fully understood [16].

Considering the lack of information on the predictors of death due to dengue in the state of Amazonas and the importance of this information for decision making and the implementation of effective measures for patient care, this study aimed to identify factors associated with the occurrence of death among patients who presented with severe dengue in the Amazonas.

## Materials and Methods

The state of Amazonas is located in the northern region of Brazil and has a total area of 1,559,161 km^2^. It is formed by 62 municipalities and 9 health regions. In 2010, the state had 3,480,937 inhabitants, of whom 1,802,525 resided in the capital city of Manaus [17].

Data on dengue cases were obtained from the SINAN (Notifiable Diseases Information System) database. Information about mortality was obtained from the SINAN and SIM (Mortality Information System) databases, provided by the FVS (Health Surveillance Foundation) of the SUSAM (Health Secretariat of the State of Amazonas).

A retrospective cohort study was conducted by using secondary data from the epidemiologic surveillance of dengue in the state of Amazonas. This study was approved by the Institutional Review Board of the Adriano Jorge Foundation Hospital, under protocol number 1162956 (CAAE no. 47148715.4.0000.0007), on July 7, 2015. This institutional review board waived the need for written informed consent from participants as the study involved only secondary data and the confidentiality of the patients’ identities was protected.

Cases of severe dengue with laboratory confirmation were selected (positive test in IgM serology, NS1 rapid test or enzyme-linked immunosorbent assay, virus isolation, polymerase chain reaction, or immunohistochemistry) and dengue-related deaths of residents in the state of Amazonas, regardless of sex or age, were recorded during the period from January 1, 2001, to December 31, 2013. Any duplicate records or incomplete data were excluded from the analysis.

We considered as severe dengue those confirmed cases recorded by SINAN presenting with the “final classification” variable filled out as “dengue with complications—DC”, “dengue hemorrhagic fever—DHF”, or “dengue shock syndrome—DSS”. In order to deal with dengue cases that do not fill WHO criteria for DHF and are not classical dengue as well, Ministry of Health (MoH) adopted the “dengue with complications” classification that is characterized by presenting at least one of the following clinical and laboratory changes: cardio-respiratory dysfunction, liver failure, gastrointestinal bleeding, neurological abnormalities, a leukocyte count equal to or less than 1,000 cells/ml, a platelet count less than 20,000 cells/mm^3^, pleural effusion, pericardial effusion or ascites. DHF is characterized by the following manifestations: high fever, hemorrhagic phenomena, thrombocytopenia, and plasma leakage. The DSS cases are characterized by all of the four criteria for DHF, plus evidence of circulatory failure manifestation. The classification of severe cases followed the criteria established by the Ministry of Health during the study period [13].

Comparing the WHO 2009 guidelines and MoH classification of “dengue with complications”, we can see that the clinical changes pointed out could be accepted as severe organ involvement. So we decided to exclude the patients classified as “dengue with complications” that present exclusively laboratorial findings like “leukocyte count equal to or less than 1,000 cells/ml” or “platelet count less than 20,000 cells/mm^3^”. This way, severe dengue definition used here met WHO 2009 criteria.

Cases considered to be deaths due to dengue were those reported to SINAN database with the “progress” variable filled out as “death due to dengue,” or from SIM database records where the underlying cause of death was filled out with the code “A90” or “A91,” according to the 10th International Classification of Diseases (ICD-10). So, dengue deaths were considered in two situations: (i) cases reported in SINAN as deaths due to dengue (n = 60) and (ii) cases registered in SIM with dengue as the underlying cause of death, but no death reported in SINAN (n = 1). It was not considered as dengue deaths: (a) SIM records that had dengue as the underlying cause of death, but not notified as dengue case in SINAN (n = 7) and (b) dengue cases reported in SINAN who died, but not due to dengue (n = 3).

The explanatory variables analyzed were sex, age, level of education, occurrence of spontaneous hemorrhagic manifestations (epistaxis, gingivorrhagia, petechiae, hematuria, gastrointestinal bleeding), platelet count (lower platelet count during hospitalization), and plasma extravasation, evidenced by the occurrence of at least one of the following signals: hemoconcentration (increase by 20% from baseline hematocrit at the time of patient admission), body cavity effusion (detected by physical examination or radiography), or hypoproteinemia (serum albumin lower than 3.0 mg/dL). These signs, symptoms, laboratory findings and therapeutic procedures were written down in the reporting and investigation forms of dengue cases according to clinical management guidelines of the Brazilian MoH [13].

Prior exploratory analysis examined the relationship between predictors and possible dengue death outcome by univariate approach. A multiple logistic regression model was used to identify independent relationships between demographic and clinical characteristics of the patients and the outcome among severe cases. Using a stepwise approach, explanatory variables were selected if they were associated with the outcome at a level of significance of 0.2. In order to reach the final model prediction, we elected the explanatory variables that remained associated with the outcome with a 5% significance level when considered simultaneously [18]. In this manner, adjusted odds ratios were calculated for each studied variable. Area under the ROC curve (AuROC) was calculated in order to estimate clinical performance of the predictive model. To perform the analysis, we used the STATA statistical package version 13 (StataCorp, College Station, Texas, USA).

## Results

**Fig 1:**
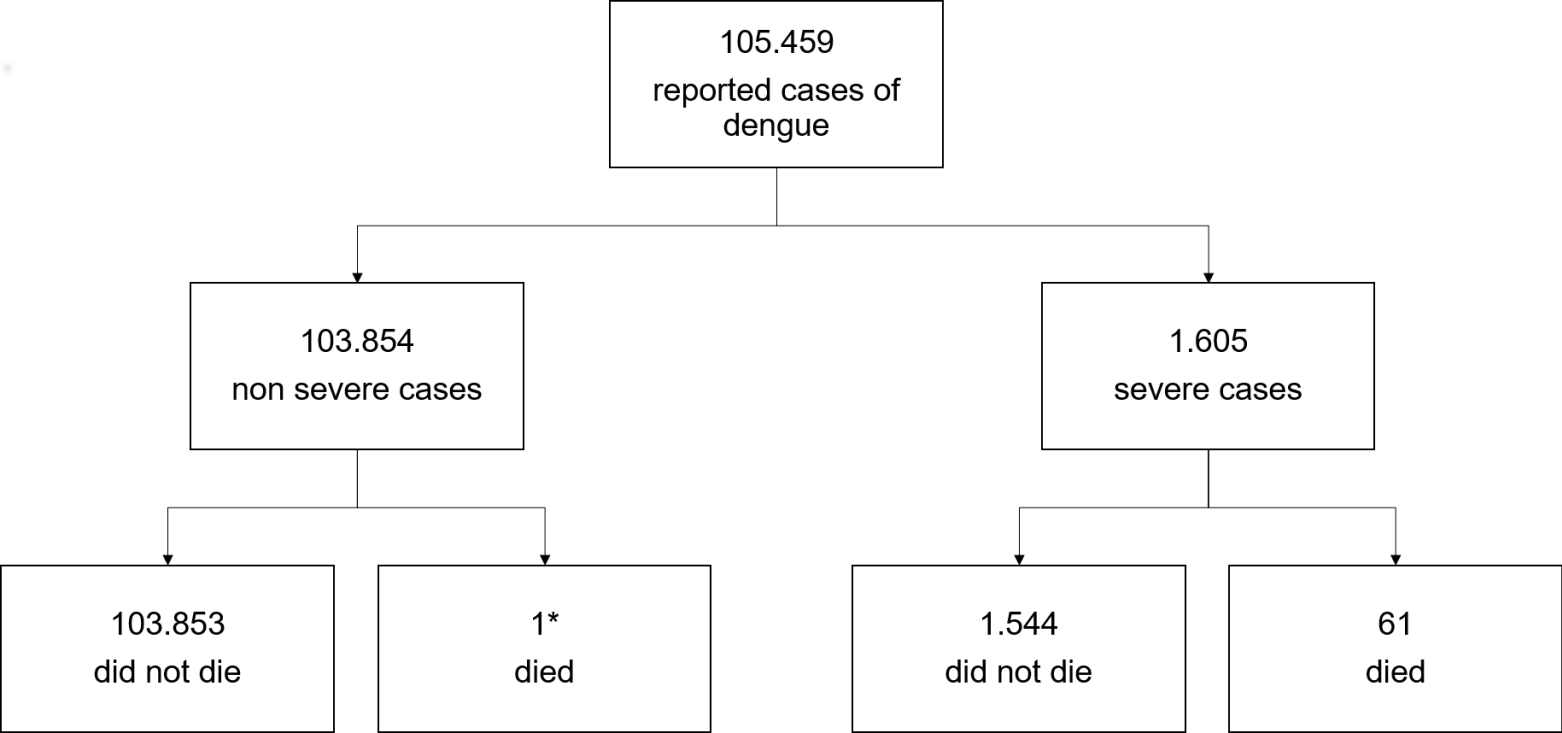
Number of dengue cases and deaths due to dengue in the years 2001 to 2013 in the state of Amazonas).

From January 1, 2001, to December 31, 2013, 105,459 cases of dengue with clinical and laboratory confirmation were reported. Of these, 1,605 (1.5%) were categorized as severe dengue. During this period, 62 deaths from dengue were recorded, of which 61 cases (98%) were diagnosed as severe cases with laboratory confirmation, and 1 case (2%) did not meet the classification criteria for severe cases (Fig 1).

Severe dengue cases occurred predominantly in young women (15–55 years old) with a level of education of >4 years schooling (Table 1). Concerning day of illness, patients present to the hospital on average on ninetieth day of illness, mode equal to four days and the range from 0 to 34. The most common clinical signs were epistaxis, petechiae, and plasma extravasation. Patients who died had more plasma extravasation, gastrointestinal bleeding, petechiae, and thrombocytopenia (Table 1).

**Table 1:** Descriptive characteristics of severe dengue cases reported in the state of Amazonas, according to the occurrence of deaths in the period from 2001 to 2013.

Characteristics	Death cases		Non-death cases		Total	
	Total number (%)		Total number (%)		Total number (%)	
**Demographic**						
Sex						
Female	33 (3.9)		817 (96.1)		850 (100)	
Male	28 (3.7)		727 (96.3)		755 (100)	
Age						
<15 years	20 (2.7)		729 (97.3)		749 (100)	
15–55 years	32 (4.1)		752 (95.9)		784 (100)	
>55 years	9 (12.5)		63 (87.5)		72 (100)	
Years of schooling						
≤4 years	7 (2.5)		277 (97.5)		284 (100)	
>4 years	20 (4.2)		460 (95.8)		480 (100)	
Not applicable^a^	9 (3.6)		244 (96.4)		253 (100)	
Ignored/missing	25 (4.3)		563 (95.7)		588 (100)	
**Clinical**						
Epistaxis						
No	37 (10.2)		327 (89.8)		364 (100)	
Yes	8 (0.9)		917 (99.1)		925 (100)	
Ignored/missing	16 (5.1)		300 (94.9)		316 (100)	
Gingivorrhagia						
No	38 (3.7)		991 (96.3)		1.029 (100)	
Yes	7 (2.7)		250 (97.3)		257 (100)	
Ignored/missing	16 (5.0)		303 (95)		319 (100)	
Petechiae						
No	22 (3.3)		640 (96.7)		662 (100)	
Yes	22 (3.4)		616 (96.6)		638 (100)	
Ignored/missing	17 (5.6)		288 (94.4)		305 (100)	
Hematuria						
No	28 (2.5)		1.098 (97.5)		1.126 (100)	
Yes	18 (12.2)		130 (87.8)		148 (100)	
Ignored/missing	15 (4.5)		316 (95.5)		331 (100)	
Gastrointestinal bleeding						
No	17 (1.6)		1.047 (98.4)		1.064 (100)	
Yes	28 (13.0)		188 (87.0)		216 (100)	
Ignored/missing	16 (4.9)		309 (95.1)		325 (100)	
Plasma extravasation						
No	14 (2.9)		477 (97.1)		491 (100)	
Yes	35 (4.0)		832 (96.0)		867 (100)	
Ignored/missing	12 (4.9)		235 (95.1)		247 (100)	
Platelet count						
≥20,000 cells/mm^3^	39 (3.2)		1.175 (96.8)		1.214 (100)	
<20,000 cells/mm^3^	22 (5.6)		369 (94.4)		391 (100)	

^a^ Patients younger than 7 years.

In the univariate analysis, among the demographic characteristics studied, the only characteristic associated with the occurrence of death is age >55 years. Gastrointestinal bleeding, hematuria, and thrombocytopenia were the clinical manifestations related to death (Table 2).

**Table 2:** Relationship between deaths due to severe dengue and demographic and clinical factors in the state of Amazonas, in the period from 2001 to 2013.

Factors	Univariate analysis			Multivariate analysis (final model)		
	Crude OR	95% CI		Adjusted OR	95% CI	
**Demographic**						
Sex						
Female	1	Reference value		-	-	
Male	0.95	0.57–1.59		-	-	
Age						
≤55 years	1	Reference value		1	Reference value	
>55 years	4.06	1.91–8.60		4.98	1.78–13.87	
Years of schooling						
≤4 years	1	Reference value		-	-	
>4 years	1.72	0.72–4.13		-	-	
**Clinical**						
Epistaxis						
No	1	Reference value		-	-	
Yes	0.99	0.49–2.00		-	-	
Gingivorrhagia						
No	1	Reference value		-	-	
Yes	0.73	0.32–1.65		-	-	
Petechiae						
No	1	Reference value		-	-	
Yes	0.9	0.54–1.51		-	-	
Hematuria						
No	1	Reference value		1	Reference value	
Yes	5.41	2.91–10.09		5.07	2.54–10.09	
Gastrointestinal bleeding						
No	1	Reference value		1	Reference value	
Yes	9.21	4.9–17.16		10.26	5.32–19.76	
Plasma extravasation						
No	1	Reference value		-	-	
Yes	1.42	0.76–2.68		-	-	
Platelet count						
≥20,000 cells/mm^3^	1	Reference value		1	Reference value	
<20,000 cells/mm^3^	1.79	1.05–3.02		2.55	1.33–4.89	

Considering the simultaneous effects of demographic and clinical characteristics by using a multiple logistic regression model, it was observed that the factors associated with death were also age >55 years, hematuria, gastrointestinal bleeding, and thrombocytopenia. Gastrointestinal bleeding was the clinical sign most strongly associated with death, followed by hematuria and age >55 years (Table 2). The model algorithm is as follows: -4.945+1.605*[age>55] + 1.623*[hematuria] + 2.328*[GI- bleeding] + 0.938*[thrombocytopenia]. In this model, an adjusted R^2^ equal to 0.22 was obtained. The final model seems to have a good performance since AuROC (c statistic) was 0.843. Models with reasonable prediction capacity have values greater than 0.7 and strong prediction capacity when more than 0.8 [19].

## Discussion

The clinical signs that were able to predict death from severe dengue among patients diagnosed with the disease in the state of Amazonas between 2001 and 2013 were gastrointestinal bleeding, hematuria, and platelet count. Age was also a factor that predicted an increased risk of death, as patients with severe dengue who were older than 55 years had a higher possibility to die of the disease.

Unfortunately, dengue remains one of the leading causes of death in developing countries, although both the infection of the virus and the evolution of the disease to death could be prevented. In the study population, the mortality rate of severe dengue was 3.8%. This rate is higher than the level desired by the Ministry of Health, whose goal is to reduce the mortality of severe cases to <1% [20]. It is noteworthy to highlight that the main strategy to reduce dengue mortality is the early identification of warning signs and appropriate clinical management of patients with a severe form of the disease.

Paixão *et al*. indicated that the risk of death caused by dengue in Brazil increased significantly between 2000 and 2011 in all regions of the country, and that both mortality and fatality rates were higher in the last year, during one of the greatest epidemics recorded in Brazil [7]. These authors questioned whether reducing the fatality rate of severe dengue in Brazil to <1% is indeed possible, given the current level of therapeutic knowledge and the capacity to accurately estimate the fatality rate. However, we believe that deaths due to dengue might be prevented by adopting appropriate clinical management. On the other hand, the occurrence of deaths due to dengue is an indicator of the weakness in health-care networks, which needs immediate attention [6].

The most common characteristics in severe dengue cases were not associated with death due to the disease. Whereas patients with severe dengue were mostly women younger than 55 years, and the most prevalent clinical signs were the appearance of petechiae, epistaxis, and plasma extravasation, the characteristics associated with death due to dengue were age >55 years, gastrointestinal bleeding, hematuria, and platelet count <20,000 cells/mm^3^.

Figueiró *et al*. analyzed the degree of actions and health services implementation, as well as the technical and scientific quality of care given to patients who died of dengue in the public health-care network of two municipalities in northeastern Brazil. The authors concluded that the occurrence of death was directly related to the clinical management of the cases, that patient care has not reached the level expected in any of the evaluated services, and that the recommendations from the Ministry of Health for the management of dengue cases are not being followed. According to the authors, the warning signs of dengue and those of shock due to the disease are not routinely investigated, and professionals have not used the clinical classification as recommended by the Ministry of Health [21].

In the same direction, the investigation of 94 deaths, conducted by the Ministry of Health in 2010, showed that aspects related to the organization of the services appear to be determining factors for the occurrence of deaths. These include a low participation of primary care as the preferred entry door of the system, the need to seek care in more than two health centers, and the lack of recognition of warning signs [22].

The importance of screening approaches and clinical management that can identify severe cases in patients seeking health services for a diagnosis, particularly those at a higher risk of complications and worse prognosis, has been already recognized. Passos *et al*. reported their experience of real-time monitoring of severe cases of dengue in Manaus during the 2011 epidemic, in which they found that clinical support led to the effective management of cases. The authors concluded that the rapid dissemination of strategic information for the prevention and control of dengue, as well as the real-time monitoring of severe dengue patients in health facilities in Manaus, allowed carrying out the appropriate management of the patients in a timely manner. Consequently, there were fewer deaths than what was expected in other previous outbreaks [23].

In our study, patients aged >55 years were more likely to die than younger patients. This finding corroborates those of other studies conducted in Brazil and other countries. Moraes *et al*. studied 12,321 cases of severe dengue reported between 2000 and 2005 in Brazil, and concluded that patients >50 years old are two times more likely to die. However, it is noteworthy to highlight that the largest epidemics occurred after that study period [24]. Garcia-Rivera and Rigau-Pérez pointed out that the elderly are at a greater risk of death during dengue epidemics, owing to the association with other morbidities that are more prevalent in this age group [25].

Our findings did not indicate any association between level of education and death due to dengue. This result can be attributed to the statistical power of the study, as well as to the occurrence of two major epidemics in the state of Amazonas. It should be noted that in a case-control study conducted in Brazil, it was found that patients with <4 years of education were 1.83 times more likely to die than those with higher levels of education [24].

In this study, patients with severe dengue had no increased risk of death in relation to sex, whereas another Brazilian study reported that women were less likely to die. It is important to note that, in our study, we considered as severe cases of dengue only those that were reported with laboratory confirmation, whereas Moraes *et al*. also included severe cases that did not meet the criteria of the Ministry of Health, i.e., “dengue with complications,” “hemorrhagic dengue Fever,” or “dengue shock syndrome” [24].

In this study, gastrointestinal bleeding, hematuria, and thrombocytopenia were identified as the clinical markers of risk of death due to dengue. Gastrointestinal bleeding was the clinical sign most strongly associated with an increased risk of death. This association was not found in the case-control national data from 2000 to 2005, which reinforces the importance of developing prediction models in specific epidemiological settings. Another possible explanation may be the greater attention given to prediction signs in the epidemics of recent years, the period during which our study took place. Severe dengue cases presenting with hematuria had a five times greater risk of death than the other cases. The association between clinical signs related to bleeding has been found to be plausible and already accepted by many researchers [26,27]. Another important finding of our study was that thrombocytopenia is a risk factor for death due to dengue. This result confirms the importance of using a cutoff point for platelet count of between 50,000 and 100,000 cells/mm^3^ as a criterion for the hospitalization of patients with dengue, as also discussed by other authors [24,28].

Our study indicates that, among deaths due to dengue that were reported in the Amazonas, a high proportion (88%) had been previously identified by health services as cases of severe disease. Thus, more careful attention to the signs of dengue could contribute to reducing the mortality of the disease.

It is known that the timely identification of dengue cases is fundamental for decision making and the implementation of prevention and control measures, with the aim to mainly prevent deaths. The effective organization of health services, in an area of epidemiological surveillance and assistance, is essential for reducing the mortality caused by dengue, as well as to allow knowing the status of the disease in each area.

This study has some limitations, mainly related to the analysis of secondary data. Underreporting of dengue cases is a reality in Brazil and, with regard to severe cases, there is a need for improvement in the proper filling out of report forms. There is also a need for improvement in the closing of cases by SINAN, as well as the proper recording of deaths by SINAN and SIM. It is likely that incorrect classifications of severe cases occurred, and the lack of laboratory confirmation might have influenced the formation of the study sample. We chose to include only cases that were confirmed by laboratory tests, as the reporting of cases during the epidemics might increase the overall number of incorrectly classified dengue cases. Regarding the missing data problem, it is difficult to assess whether there was selective withdraw or losses were by random. We assessed whether individuals with missing and complete data for the main predictors were different in relation to sex and age. The results indicate that there is no statistically significant difference between groups (data not shown). These findings weaken the plausibility of selection bias due to missing data.

This study sheds light on the individual and clinical characteristics that can predict death among patients with severe dengue, in the historical and epidemiological contexts of the disease in which health services need to prepare the best surveillance strategies in order to reduce mortality to the lowest possible levels.

In conclusion, considering our findings, the best prediction of death among severe dengue cases could be done based on individual characteristic of older age (>55 years), simultaneously with clinical signs of gastrointestinal bleeding, hematuria, and thrombocytopenia. This prediction model can be used both in improving the clinical management of severe dengue and to adopt specific surveillance strategies to recognize individuals with higher risk of death during episodes of dengue to be diagnostic in forthcoming epidemics. So the external validity of this prediction model could be performed in this moment. Confronting new epidemics could be benefited by training clinicians and other professionals who handle severe dengue using locally recognized predictors rather than those identified in clinical and epidemiological settings distinct from that observed in the Amazon.
